# Identification of an ACK1/TNK2-based prognostic signature for colon cancer to predict survival and inflammatory landscapes

**DOI:** 10.1186/s12885-021-09165-w

**Published:** 2022-01-20

**Authors:** Defeng Kong, Guoliang Li, Zhenrong Yang, Shujun Cheng, Wen Zhang, Lin Feng, Kaitai Zhang

**Affiliations:** 1 State Key Laboratory of Molecular Oncology, Department of Etiology and Carcinogenesis, National Cancer Center/National Clinical Research Center for Cancer/Cancer Hospital, Chinese Academy of Medical Sciences and Peking Union Medical College, Beijing, 100021 PR China; 2 Department of Immunology, National Cancer Center/National Clinical Research Center for Cancer/Cancer Hospital, Chinese Academy of Medical Sciences and Peking Union Medical College, Beijing, 100021 PR China

**Keywords:** ACK1, Colon cancer, Prognosis, Immune infiltration

## Abstract

Activated Cdc42-associated kinase 1 (ACK1), a kind of tyrosine kinase, is considered to be an oncogene in many cancers, and it is likely to become a potential target for cancer treatment. We found that the expression of the ACK1 gene in colon cancer was higher than that in normal tissues adjacent to cancer, and high expression of the ACK1 gene was associated with poor prognosis of patients. We assessed the prognosis of colon cancer based on ACK1-related genes and constructed a model that can predict the prognosis of colon cancer patients in colon cancer data from The Cancer Genome Atlas (TCGA) database. We then explored the relationship between ACK1 and the immune microenvironment of colon cancer. The overexpression of ACK1 might hinder the function of antigen-presenting cells. The colon cancer prognosis prediction model we constructed has certain significance for clinicians to judge the prognosis of patients with colon cancer. The expression of the ACK1 gene might affect the infiltration level of a variety of immune cells and immunomodulators in the immune microenvironment.

## Background

Colon cancer is a major health burden worldwide, and the incidence of colon cancer is on the rise [5]. At present, the treatment of colon cancer is mainly based on traditional treatments such as surgery, radiotherapy and chemotherapy. With in-depth research on the mechanism of colon cancer occurrence and development in recent years, many new treatment methods have been discovered, including molecular targeted therapy and immunotherapy, but these treatment methods have limited efficacy.

Until recently, the gene targets of precision therapy drugs mainly included MSI [30], BRAF [1], KRAS [7], NRAS [21], HER2 [10], NTRK [23], etc. However, these findings still cannot meet the needs of the many colon cancer patients with different molecular types. Therefore, more effective therapeutic targets and more precise molecular classification of colon cancer need to be explored.

With the continuous progress of immunotherapy, it is necessary to establish reliable biomarkers for immune guidance. By inferring markers that are sensitive to immunotherapy, broadening our understanding of overlapping disease molecular fragments may help to better identify patients who respond to immunotherapy or targeted therapy [2].

ACK1 tyrosine kinase is abnormally activated, amplified or mutated in a variety of human cancers. Dysregulated kinase is carcinogenic, and its activation is related to the metastatic stage. The carcinogenicity of ACK1 is not only due to its ability to promote the activation of key presurvival kinases and receptors by phosphorylation of different tyrosine residues but also due to the use of similar mechanisms to eliminate tumour suppressors in cancer cells. ACK1/TNK2 is a nonreceptor tyrosine kinase (NRTK) that represents a paradigm of tyrosine kinase signalling and seems to be addictive to cancer cells. Since the ACK1 signal can be activated by multiple ligands in the same cell, its importance is further emphasized. This finding is particularly important in cancers that are resistant to the inhibition of a single RTK pathway and have activated alternative RTK-regulated pathways to survive [14, 16].

There has been almost no research on the role of ACK1 in colon cancer, especially in terms of immune invasion and prognosis of colon cancer. Therefore, this article explored the use of ACK1 to infer the prognosis of colon cancer and the immune microenvironment. ACK1 may become a potential target for precision therapy that benefits colon cancer patients.

## Methods and materials

### Data sources and access to clinical information

This article selected the colon adenocarcinoma (COAD) cohort in the TCGA database. The clinical information for these patients was also downloaded directly from the TCGA database. Additionally, we selected two sets of data from the Gene Expression Omnibus (GEO) (https://www.ncbi.nlm.nih.gov/geo/query/acc.cgi?acc=GSE9348, GSE44076). The immunohistochemical pictures provided in the article were from the Human Protein Atlas available from http://www.proteinatlas.org [27].

### Gene function enrichment analysis

Gene ontology (GO) and Kyoto Encyclopedia of Genes and Genomes (KEGG) were implemented using R software version 4.0.2 and the R package “clusterProfiler”.

### Signature identification based on ACK1-related genes and establishment of a prognostic model

We selected the top 500 genes associated with ACK1 with *P* < 0.05. Then, the least absolute shrinkage and selection operator (LASSO)-Cox model with the “glmnet” and “survival” R packages was used to screen variables, and 8 genes were used to construct a prognostic model. The risk score was calculated: Risk score = (Gene1 * coef Gene1) + (Gene2 * coef Gene2) + … + (Gene8 * coef Gene8). All patients were divided into two groups according to the risk score. The survival analysis of the high-risk group and the low-risk group was analysed by the Kaplan–Meier method and two-tailed log-rank test, and *P* < 0.05 was considered to be significantly different. We used nomograms combined with clinical characteristics and patient risk scores for cancer prognosis. The nomogram was created by the rms package of R software. The consistency index (C-index) was used to measure the prediction accuracy of the nomogram. In addition, we randomly split the TCGA colorectal cancer dataset at a ratio of 7:3 for internal verification.

### Analysis of immune cell infiltration in colon cancer

Cell type identification by estimating relative subsets of RNA transcripts (CIBERSORT) was adopted to qualify and quantify 22 types of immune cells in colon cancer tissues. (https://cibersort.stanford.edu/) [19]. The results were displayed using R software version 4.0.2.

### The relationship between ACK1 and immunomodulators

The correlation analysis between genes and immune cells was processed by the Tumour Immune Estimation Resource (TIMER) database (cistrome.dfci.harvard.edu/TIMER/) [13], and the correlation analysis between genes and immunomodulators was performed on the TISIDB website (http://cis.hku.hk/TISIDB/) [20].

### Statistical analysis

Statistical analysis was performed using R software version 4.0.2. In all statistical analyses presented in this article, when *P* < 0.05, the difference was considered statistically significant.

## Results

### ACK1/TNK2 is differentially expressed in colon cancer and adjacent tissues

**Fig. 1:**
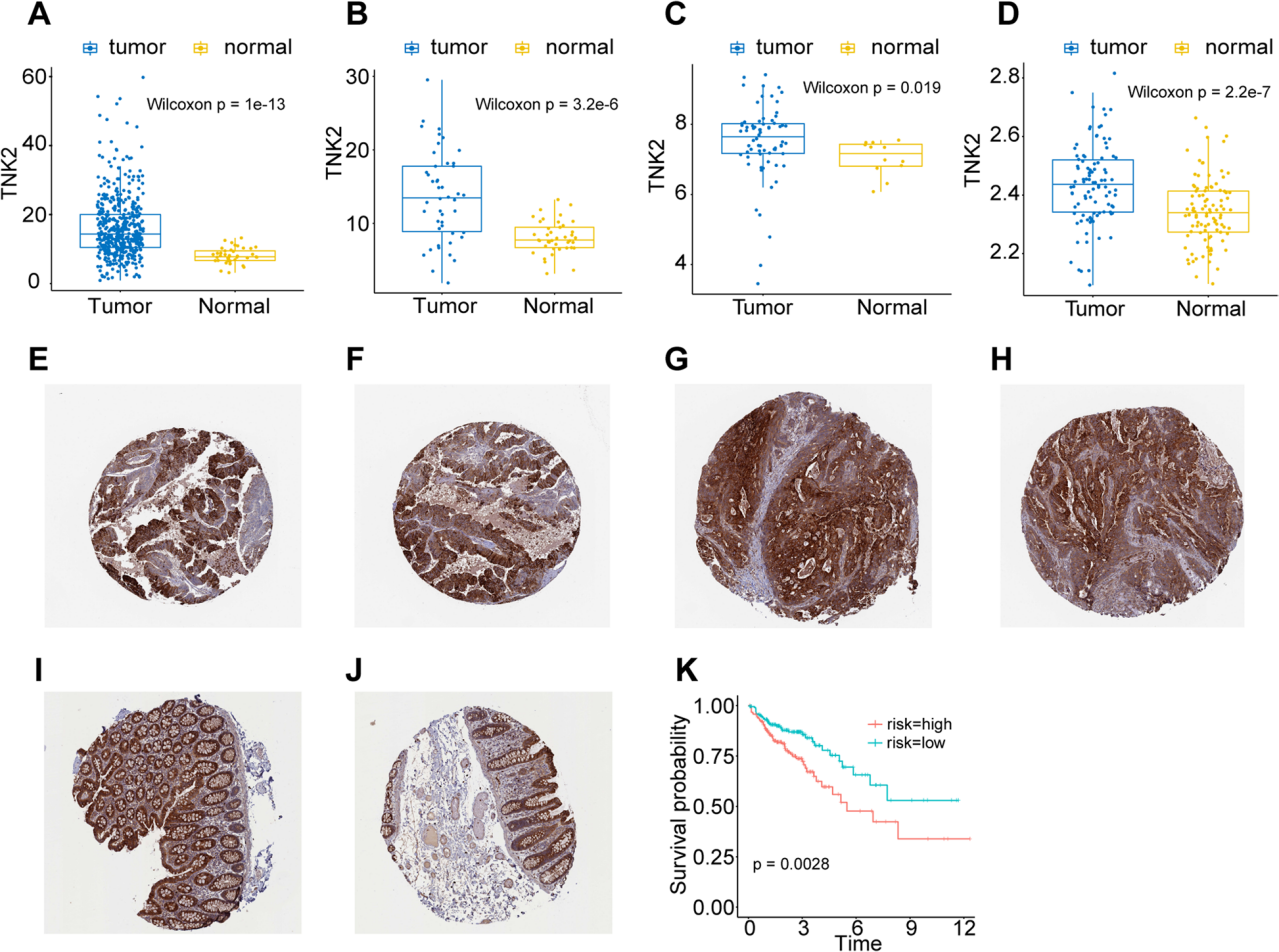
Expression of ACK1/TNK2 gene and protein in cancer tissues and normal tissues adjacent to cancer and its relationship with prognosis. **A** TCGA cohort; **B** Paired samples of TCGA cohort; **C** Data from GSE9348; **D** Data from GSE44076; **E**, **F**, **G**, **H** Immunohistochemical results of ACK1 in colon cancer tissue from HPA database. **E** HPA041954; **F** HPA041954; **G** HPA041954; **H** HPA041954; **I**, **J** Immunohistochemical results of ACK1 in normal tissues adjacent to cancer from the HPA database. **I** HPA041954; **J** HPA041954. **K** Survival curve of ACK1 expression in the TCGA colon cancer cohort

We adopted colon cancer data from the TCGA database. In the colon cancer cohort, ACK1 expression in cancer tissues was higher than that in adjacent cancer tissues (Fig. 1A, B). We also verified the results in two independent datasets from the GEO database (Fig. 1C, D). This provided a chance for ACK1 become one of the targets of colon cancer treatment. Colon cancer patients were divided into two groups according to the expression of ACK1. The prognosis of patients in the ACK1 high expression group was worse (Fig. 1K).

We referred to immunohistochemical images of colon cancer tissue and normal colon tissue from the Human Protein Atlas, and the results showed that the expression level of ACK1 protein was higher in colon cancer tissue (Fig. 1E-J) [27].

### Signal pathway analysis of ACK1 related genes

**Fig. 2:**
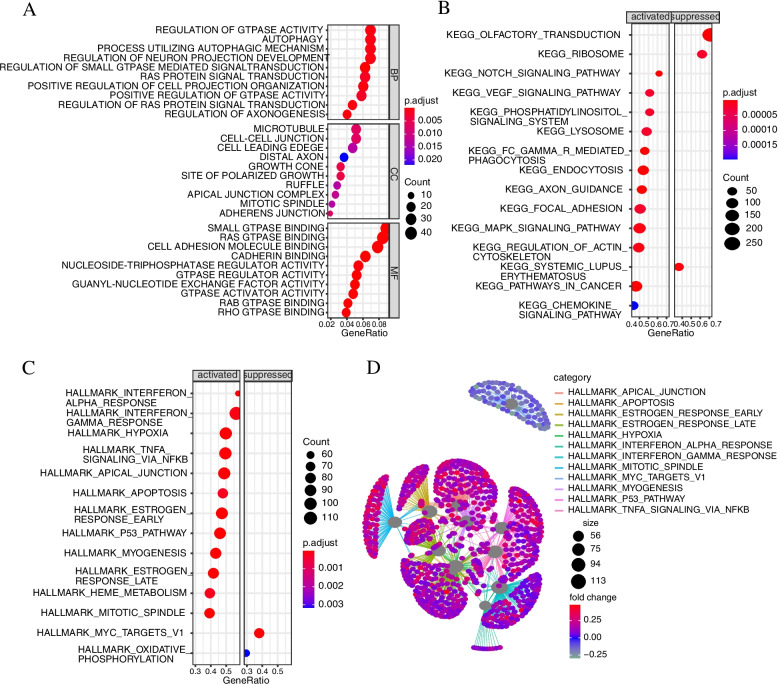
Gene pathway enrichment analysis of ACK1/TNK2-associated genes. **A** Gene Ontology annotation. **B** Kyoto Encyclopedia of Genes and Genomes pathway analysis. **C** Gene Set Enrichment Analysis. **D** Net-plot of gene enrichment analysis

To study the signal regulation characteristics of genes related to ACK1, we screened the top 500 genes related to ACK1 and performed signal pathway enrichment analysis. The activated signalling pathways included the following cancer-driving gene signalling pathways: P53 pathway, inflammatory signalling pathway interferon alpha pathway, interferon gamma pathway, hypoxia signalling pathway, TNFα signalling pathway, ras protein signal transduction, autophagy, oestrogen response pathway, apoptosis pathway and oxidative phosphorylation signalling pathway. These signalling pathways play a vital role in the occurrence and development of cancer. Some of these signalling pathways have become classic target pathways for cancer targeted therapy, such as the ras protein signal transduction pathway and oestrogen response pathways (Fig. 2). This result indicated that ACK1 might be a potential target for cancer targeted therapy.

### A prognostic model based on ACK1-related genes can be used as an indicator to evaluate the prognosis of colon cancer

**Fig. 3:**
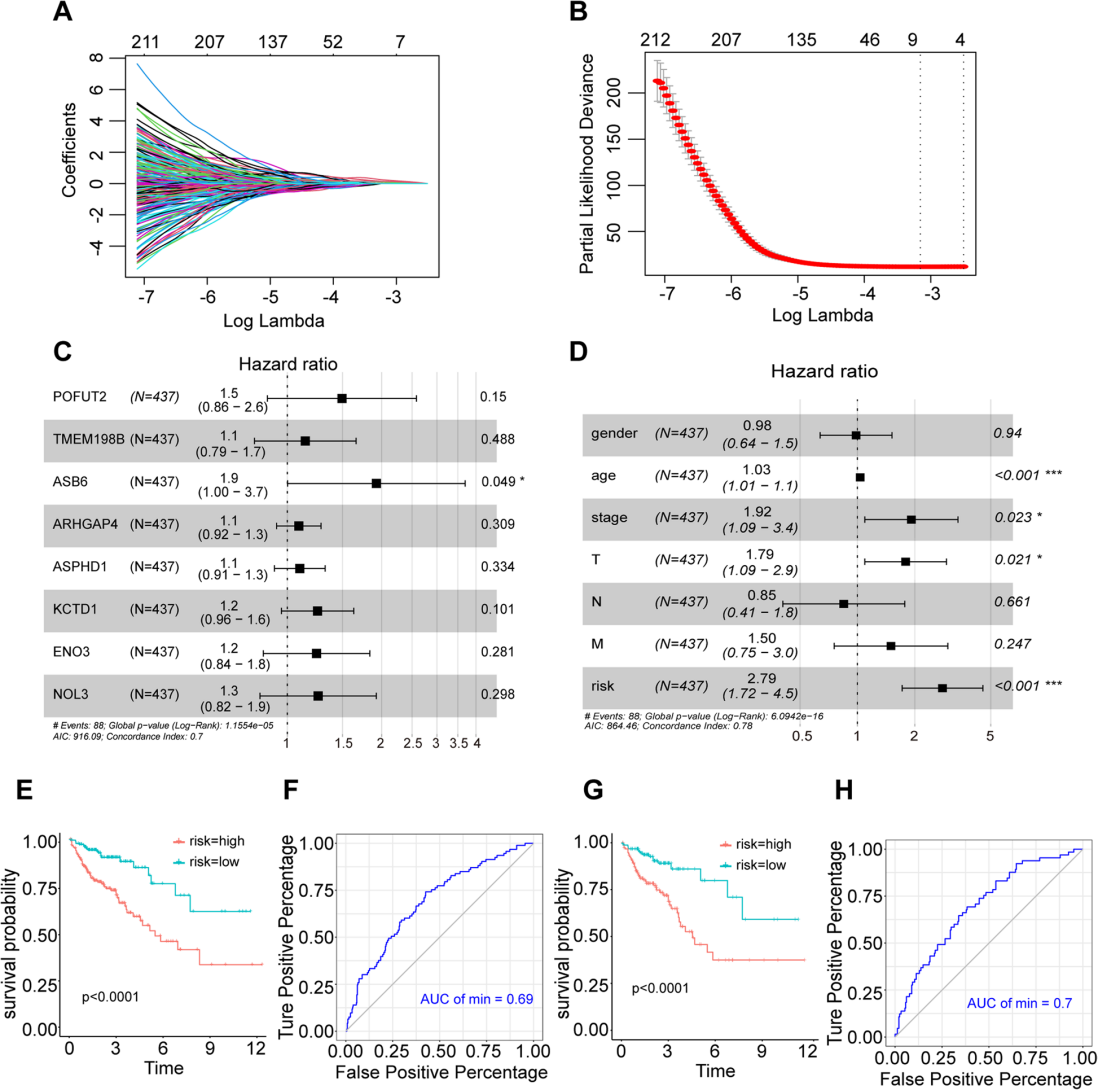
An 8-gene prognostic model based on ACK1/TNK2-associated genes. **A** The LASSO coefficient profiles of the most useful prognostic genes. **B** Plot of cross-validated partial likelihood deviances. The number on the top of the plot shows the number of genes of each model. **C** Results of the multivariate Cox regression analyses of genes in the model regarding OS in the COAD cohort. **D** Results of the univariate and multivariate Cox regression analyses of clinical features and risk of model regarding OS in the COAD cohort. **E** Prognostic analysis of high- and low-risk groups according to the risk score of the 8-gene prediction model. **F** ROC curve of 8 gene prediction model. **G** Prognostic analysis of internal validation set. **H** ROC curve of internal validation set

We constructed a prognostic model with eight genes (POFUT2*0.129542673119284 + TMEM198B*0.097065434827916 + ASB6*0.220764646390914 + ARHGAP4*0.00935357399314663 + ASPHD1*0.0140944673936701 + KCTD1*0.0849497715526077 + ENO3*0.134401085942573 + NOL3*0.1370027521824) based on ACK1-related genes (Fig. 6A, B, C). According to our model, patients were divided into high and low groups by calculating risk scores, and the prognosis of patients in the high-risk group was worse (Fig. 3E). The risk score was significantly associated with survival in COAD, as indicated by the multivariate Cox regression analyses (HR = 2.79, 95% CI = 1.72–4.5, *P* < 0.001) (Fig. 3D). The area under the curve (AUC) of the receiver operating characteristic curve (ROC) was 0.69 (Fig. 3F). Subsequently, we conducted internal verification of the randomly selected data from the TCGA colon cancer dataset. The patients in the high-risk group had a worse prognosis (Fig. 3G), and the AUC of the ROC curve was 0.69 (Fig. 3H).

**Fig. 4:**
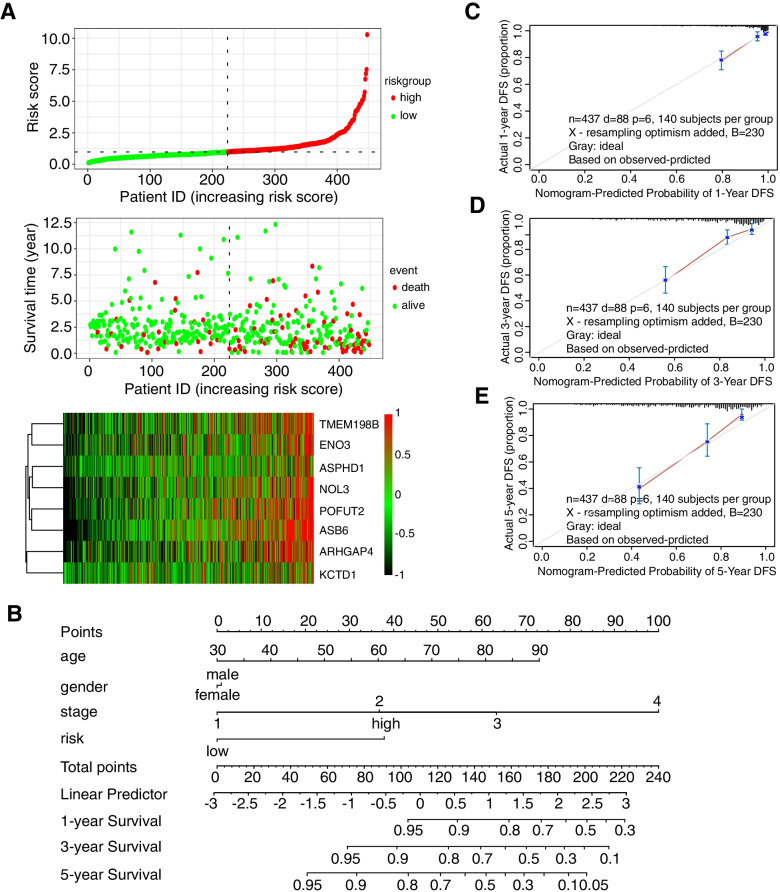
The 8-gene prognostic model and nomogram chart based on ACK1/TNK2-associated genes. **A** Distribution of risk scores, survival statuses, and gene expression profiles of genes in the model for COAD. **B** Nomogram predicting overall survival for COAD patients. **C**, **D**, **E** The calibration plots of the nomogram

We constructed a prognostic nomogram in COAD to anticipate the individuals’ survival probability by weighing risk score, stage, age, and sex. Calibration was performed for the nomogram (Fig. 4B). The calibration curve showed that the probability predicted by the nomogram was consistent with the ideal reference line for 1-year, 3-year and 5-year survival rates (Fig. 4C, D, E). We also evaluated the predicted discrimination of the nomogram using the C index, which quantifies the level of agreement between the probability derived from the nomogram and the actual death observation. The C index of our prognostic nomogram reached 0.78.

### The landscape of infiltrating immune cells in colon cancers and normal tissues

**Fig. 5:**
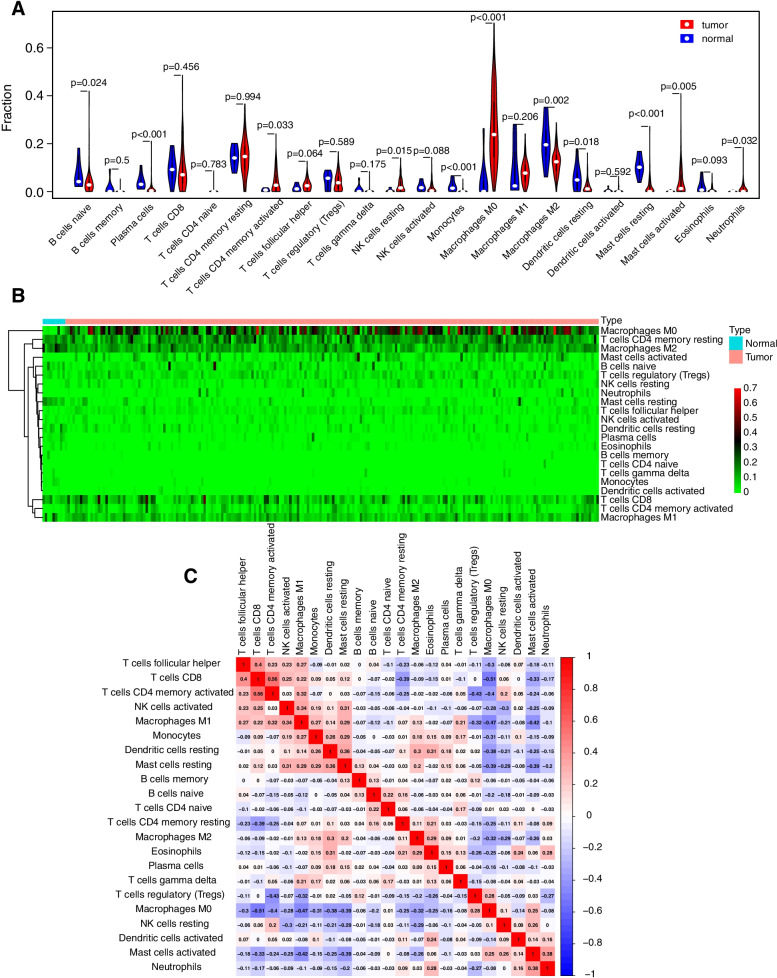
COAD immune infiltration analysis based on cell type identification by estimating relative subsets of RNA transcripts (CIBERSORT) method. **A** Violin plots showing the differences in the immune cell distribution between cancer (red) and adjacent normal tissues (blue) in COAD. **B** Heatmaps indicating the differences in the immune cell distribution between cancer (red) and adjacent normal tissues (blue) in COAD. **C** Correlation between immune cells in COAD

We systematically delineated the pattern of immune cells by extracting and processing the signature gene expression profile with the CIBERSORT method. After removing the samples with *P* ≥ 0.05, the landscape of the infiltrating immune cells in cancer tissues and adjacent cancer tissues for TCGA colon cancer cohorts is displayed in Fig. 5. Naive B cells, plasma cells, monocytes, M2 macrophages, resting dendritic cells and resting mast cells had a higher degree of infiltration in adjacent tissues. CD4 memory-activated T cells, resting NK cells, M0 macrophages, M1 macrophages, activated mast cells, and neutrophils had an increased infiltration rate in cancer tissues (Fig. 5A, B).

### ACK1 expression is related to the degree of immune cell infiltration

**Fig. 6:**
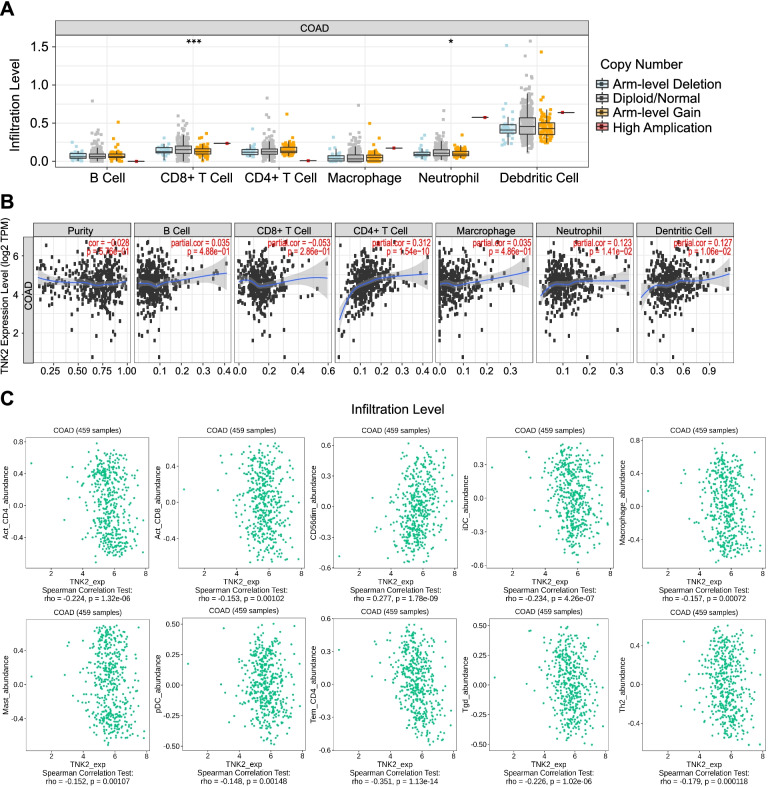
Correlation analysis between ACK1, immune cells and immunomodulators processed by the TIMER database and the TISIDB website. **A** Correlation between ACK1 gene copy numbers and immune cell infiltration levels in COAD. *p < 0.05; **p < 0.01; ***p < 0.001. **B** The correlation between the expression level of the ACK1 gene and different immune cells in COAD by the TIMER database. **C** Correlation between ACK1/TNK2 gene expression and different immune cell subgroups by the TISIDB website

Subsequently, we investigated the interaction between ACK1 gene expression and tumour immune infiltration. The immune cell infiltration levels changed along with the ACK1/TNK2 gene copy numbers. Two immune cell infiltration levels seemed to be associated with altered ACK1/TNK2 gene copy numbers, including CD8+ T cells and neutrophils, in COAD (Fig. 6A). The expression of ACK1 was also positively correlated with the infiltration levels of CD4+ T cells and dendritic cells (Fig. 6B). In a more detailed classification of immune cells, the expression of ACK1 was negatively correlated with several immune cells, including activated CD4+ T cells, activated CD8+ T cells, iDCs, macrophages, mast cells, pDCs, Tem CD4+ cells, Tgd cells and Th2 cells, but not CD56dim cells (Fig. 6C).

### ACK1 expression is related to the expression of immune checkpoint proteins

**Fig. 7:**
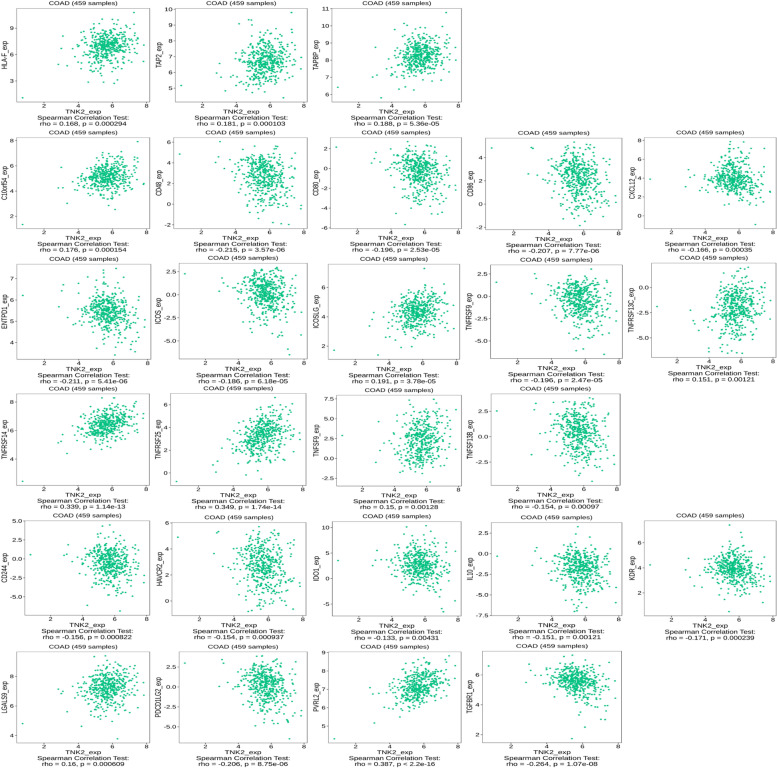
Correlation between the ACK1/TNK2 gene and immunomodulators

ACK1 was positively correlated with the expression of HLA-F, TAP2 and TAPBP (Fig. 7). These three proteins belong to the MHC family. HLA-F was negatively correlated with overall survival (OS) in all grades of glioma and glioblastoma (GBM) [9]. Abnormal function of the TAP gene plays an important role in tumorigenesis and development [12]. Then, we explored the relationship between ACK1 and immunostimulators as well as immunoinhibitors (Fig. 7). Signals initiated through both the TCR complex and CD28 were required for optimal activation of T lymphocytes. Recently, it has been demonstrated that CD28 interacts with two different ligands, designated CD80 (B7/B7–1) and CD86 (B70/B7–2). The roles of CD80 and CD86 in an immune response may be determined primarily by their differential expression on APCs [3, 11]. However, ACK1 was negatively correlated with the expression of CD80 and CD86, so we inferred that the overexpression of ACK1 might hinder the function of antigen-presenting cells. The study by Duhen and colleagues showed that CD103 + CD39+ tumour-infiltrating CD8 T cells (CD8 TILs) were enriched for tumour-reactive cells in both primary and metastatic tumours. CD103 + CD39+ CD8 TILs also efficiently killed autologous tumour cells in an MHC class I-dependent manner [8]. However, the expression of ACK1 was negatively correlated with the expression of ENTPD1. The expression of ACK1 might be detrimental to the killing function of CD8 TILs. ACK1 was positively correlated with the expression of TNFRSF14. Tumour necrosis factor receptor superfamily 14 is highly expressed in various tumour tissues and plays critical roles in tumour biology. A high level of TNFRSF14 expression was associated with poor overall survival (OS) and disease-free survival (DFS) in patients with clear cell renal cell carcinoma (ccRCC) [24].

Tumours evade immune-mediated recognition through multiple mechanisms of immune escape. During the last decade, immunotherapies targeting IRs such as programmed cell death receptor 1 (PD-1) and anticytotoxic T lymphocyte-associated antigen 4 (CTLA-4) have provided ample evidence of clinical benefits in many solid tumours. Beyond CTLA-4 and PD-1, multiple other IRs were also targeted with immune checkpoint blockade in the clinic. Specifically, the T cell immunoreceptor with immunoglobulin and ITIM domain (TIGIT) is a promising new target for cancer immunotherapy. TIGIT is upregulated by immune cells, including activated T cells, natural killer cells, and regulatory T cells. TIGIT binds to two ligands, CD155 (PVR) and CD112 (PVRL2, nectin-2), which are expressed by tumour cells and antigen-presenting cells in the tumour microenvironment [6]. The expression of ACK1 and PVRL2 was negatively correlated. Therefore, the immune microenvironment of colon cancer tissues overexpressing ACK1 may be very complicated and needs to be further explored.

## Discussion

The ACK1 gene is located on human chromosome 3q29, encodes a large protein (140 kDa) of 1038 amino acids and contains at least 8 different domains. This multidomain structure not only promotes the localization of ACK1 to different cell compartments but also promotes its association with disparate proteins, fostering its functional diversity [15].

The ACK1 gene is oncogenically activated in a variety of cancers, such as lung cancer, head & neck squamous cell carcinomas, breast cancer and gastric cancer [18, 22, 26, 31]. Aberrant ACK1 activation leading to its oncogenicity may occur by at least three distinct mechanisms: deregulated RTK activation feeding into ACK1, gene amplification and somatic missense mutations [15].

Although the role of ACK1 in promoting the occurrence and development of cancer has been found in many cancers, in colon cancer, the impact of the ACK1 gene on the immune microenvironment and the prognosis of patients has not been reported.

The occurrence and development of colon cancer is a complex process involving multiple genes and multiple stages. At present, many important driver genes have been discovered, such as P53, APC, and KRAS [28, 29]. Driver genes and accompanying genes can become targets for tumour therapy [7]. The development of cancer is a process in which tumour cells interact with the microenvironment. It is very important to study how driver genes interact with the immune microenvironment.

We found that there was a significant difference in the expression of the ACK1 gene between colon cancer tissues and adjacent normal tissues. As an oncogene, the high expression of ACK1 in tumour tissues may explain its role in tumour initiation. Therefore, ACK1 may be a potential therapeutic target [14, 17]. The expression of ACK1 is significantly related to prognosis.

The expression of a single gene may vary due to different samples or sequencing methods, so it is often impossible to accurately predict the prognosis of patients with a single gene. However, gene signature can remedy this problem. Multigene verification can reduce the deviation caused by the specificity of a single gene. A prognostic model built on the basis of ACK1-related genes can infer the patient’s prognostic status. This model provides a new method for evaluating the prognosis of colon cancer patients.

Colon cancer is highly related to inflammation. Inflammation plays an indispensable role in the process of canceration and progression of colon tissue. Inflammation causes changes in the immune microenvironment of colon tissue, and long-term chronic inflammation promotes the survival of tumour cells. In addition, inflammation leads to changes in the composition of the intestinal flora, which indirectly lead to the formation of cancer [25, 28]. The relationship between ACK1, immune cells and immunomodulators also provides a point for understanding the immune microenvironment of colon cancer.

The study by Bindea et al. reported that the density of B cells was elevated in adjacent tissues [4]. This result was consistent with our research. In general, the immune microenvironment of colon cancer is very complex and worthy of further exploration and research.

Based on the analysis of TCGA data, GEO data and protein expression data, this article found that ACK1 is more highly expressed in colorectal cancer tissues than in adjacent tissues and that patients with high ACK1 expression have a poorer prognosis. Through GO and KEGG analysis, it was found that the high expression of ACK1 is related to the P53 pathway, inflammatory signalling pathway interferon alpha pathway, interferon gamma pathway, hypoxia signalling pathway, TNFα signalling pathway, ras protein signal transduction, autophagy, oestrogen response pathway, apoptosis pathway and oxidative phosphorylation signalling pathway. This indicates that ACK1 may be a driver gene related to the occurrence and development of colon cancer and may become a therapeutic target in the future, providing a new target for targeted therapy. Subsequently, we made a prediction model for predicting the prognosis of colorectal cancer patients based on ACK-related genes. The prognosis of patients in the high-risk group is worse, which helps clinicians predict the survival time of colorectal cancer patients. Through the analysis of immune infiltration, we also found that the ACK1 gene is related to a variety of immune cells, indicating that ACK1 may be involved in the regulation of the tumour immune microenvironment, which plays a very complicated and unclear role. Therefore, it is very important to further study the relationship between ACK1 and immune cells and the immune microenvironment.

Our research also has some limitations. Although all of our results were based on a large amount of data analysis, more in-depth research on ACK1 needs to be verified by experiments in the future.

The ACK1 gene is related to many important signal transduction pathways, but its mechanism of action still needs to be experimentally verified. The clinical information included in the multivariate analysis of our model is limited and does not include information such as whether the patient had surgery or not and whether the patient received immunotherapy. In our immune cell infiltration analysis, we found that the ACK1 gene is related to a variety of immune cells, but this correlation needs to be verified by experiments, and the role of these immune cells in the occurrence and development of cancer still needs to be further explored.

## Conclusions

In conclusion, the relationship between the ACK1 gene and immunomodulators may provide a reference for the immunotherapy of colon cancer. In addition, the eight-gene prognostic model based on the correlation of the ACK1 gene will be helpful for clinicians to assess the prognosis of patients with COAD.

## Data Availability

Publicly available datasets analyzed in this study are available in the Cancer Genome Atlas (Repository (cancer.gov) ) and GEO database (Home - GEO - NCBI (nih.gov) ) (GSE9348 (GEO Accession viewer (nih.gov) ), GSE44076 (GEO Accession viewer (nih.gov) )).

## Abbreviations

ACK1: Activated Cdc42-associated kinase1
TCGA: The Cancer Genome Atlas
NRTK: Non-receptor tyrosine kinase
GEO: Gene Expression Omnibus
GO: Gene ontology
KEGG: Kyoto Encyclopedia of Genes and Genomes
LASSO: Least absolute shrinkage and selection operator
C-index: Consistency index
CIBERSORT: Cell type Identification by Estimating Relative Subsets of RNA Transcripts
TIMER: Tumor Immune Estimation Resource
AUC: Area under curve
ROC: Receiver operating characteristic curve
OS: Overall survival
GBM: Glioma and glioblastoma
PD-1: Programmed cell death receptor 1
CTLA-4: Anticytotoxic T lymphocyte-associated antigen 4
CD8 TIL: Tumor-infiltrating CD8 T cells
DFS: Disease-free survival
TIGIT: T cell immunoreceptor with immunoglobulin and ITIM domain
COAD: Colon adenocarcinoma
ccRCC: Clear cell renal cell carcinoma
